# An In Situ Evaluation of the Protective Effect of Nano Eggshell/Titanium Dioxide against Erosive Acids

**DOI:** 10.1155/2018/4216415

**Published:** 2018-12-02

**Authors:** Stanley Chibuzor Onwubu, Phumlane Selby Mdluli, Shenuka Singh, Sanele Nyembe, Rookmoney Thakur

**Affiliations:** ^1^Dental Sciences, Durban University of Technology (DUT), Durban, South Africa; ^2^Chemistry, Durban University of Technology (DUT), Durban, South Africa; ^3^Discipline of Dentistry, University of KwaZulu-Natal (UKZN), Durban, South Africa; ^4^DST/Mintek Nanotechnology Innovation Centre, Advanced Materials Division, Mintek, Private Bag X3015, Randburg, Johannesburg 2125, South Africa

## Abstract

**Objectives:**

Enamel erosion caused by high consumption of acidic drinks poses a significant public health concern. This study was aimed to determine the protective effect of eggshell-titanium dioxide composite (EB@TiO_2_) against erosive acids on tooth enamel.

**Methods:**

Twenty prepared bovine tooth enamel specimens were randomly assigned to 5 sample groups (*n*=4): (1) unexposed tooth enamel; (2) exposed tooth enamel + HCI; (3) exposed tooth enamel + HCI + Colgate toothpaste; (4) exposed tooth enamel + HCI + Sensodyne toothpaste; and (5) exposed tooth enamel + HCI + EB@TiO_2_. The mean roughness value (*R*_rms_) of the exposed and unexposed tooth was measured with atomic force microscope (AFM). Scanning electron microscope (SEM) and Raman spectroscopy techniques were used to evaluate the surface morphology and changes. ANOVA was used to analyze the mean square roughness (*R*_rms_) values for all specimens. Bonferonni correction was used to identify the mean differences among the 5 groups (*α*=0.05). The *R*_rms_ values measured for the unexposed and exposed specimens in HCI alone were statistically significant (*P* < 0.05).

**Results:**

No significant differences were found for the unexposed and exposed specimens in HCI + toothpaste and EB@TiO_2_. The tooth enamel specimens exposed to HCI + Sensodyne had the highest *R*_rms_ values, while specimens exposed to HCI + EB@TiO_2_ had the lowest *R*_rms_ values.

**Conclusions:**

This study confirms that the investigated toothpaste provides protection against acidic substances. The study results further suggests that EB@TiO_2_ could be used to provide enhanced protection for tooth enamel.

## 1. Introduction

Enamel erosion has become a topical issue in recent years among clinicians and oral health-care providers due to the increase in the consumption of acidic drinks such as soft drinks, energy drinks, and fruit drinks [1]. Previous studies [2, 3] reported that these drinks have a pH [2–4] value that facilitates the dissolution of the tooth and the destruction of the mineral composition of the enamel. Several other authors [4, 5] have demonstrated that as a result of consumption of acidic drinks, the onset of caries and enamel erosion correlate positively together.

**Figure 1:**
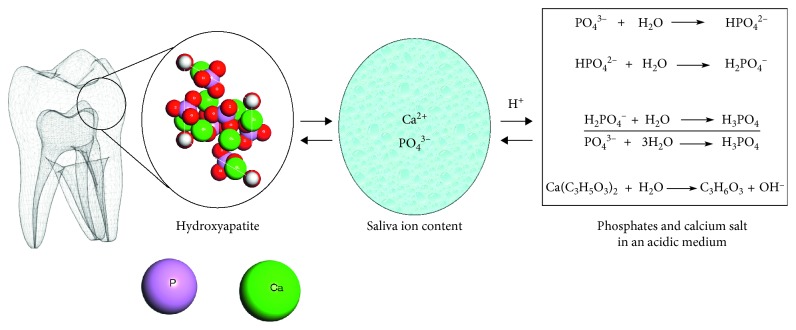
Illustration of tooth demineralization and remineralization dynamism in oral and acidic environment.

Whilst the tooth enamel is mainly composed of hydroxyapatite in the form of phosphate ions (PO_4_^3−^) and calcium ions (Ca^2+^), Shellis et al. [6], and Lussi and Carvalho [7] argued that there exists an equilibrium between the tooth enamel crystals and the surrounding oral environment. The destabilization of these equilibrium, particularly when the oral environment pH drops below a critical level (5.5 for enamel and 6.2 for dentin), may result in the dissolution of tooth mineral composition [8]. Moreover, the loss of enamel material by erosion is a dynamic process occasioned with periods of demineralization and remineralization (Figure 1); hence, preventive measures against enamel dissolution from acid and potentially permanent damage should be a priority for oral health-care providers [2, 3].

Traditionally, toothpastes have been considered effective and accessible vehicles to improve enamel resistant against erosive oral environment [9]. In recent years, different ingredients have been added to the toothpastes composition to enhance their protective effect against erosive substances. Amongst these ingredients, the use of topical fluoride to modify the effects of erosion at the tooth surface is well documented [10–13]. However, Moron et al. [14] stressed that conventional fluoride-containing toothpastes lack the capacity to protect sufficiently well against erosive substance. Furthermore, Larsen and Richards [15] revealed that at pH below 3, the protective effect of fluoride is diminished. Consequently, it has been suggested that the beneficial health effects against erosive attacks can be improved with the addition of calcium or calcium containing material into toothpaste [16].

Significantly, studies by King'Ori [17] and Onwubu et al. [18] suggest that the calcium of eggshell can be used as an abrasive cleaner for dental plaque in toothpaste. Lin et al. [19] and Tao et al. [20] equally demonstrated that the mechanical modification of calcium carbonates together with titanium dioxide improved the acid resistance properties when used in paper making industry. However, there is a limited research on the protective effect of a modified eggshell-titanium dioxide composite (EB@TiO_2_) on tooth enamel. This in vitro study aimed to determine the protective effect of a modified eggshell-titanium dioxide composite EB@TiO_2_ against erosive acids. The protective level of EB@TiO_2_ against erosive acids was compared against Colgate® and Sensodyne® toothpaste. It was hypothesized that EB@TiO_2_ will offer more protection against erosive challenge than the commercial toothpastes.

## 2. Materials and Methods

### 2.1. Preparation of Eggshell-Titanium Dioxide Composite (EB@TiO_2_)

**Figure 2:**
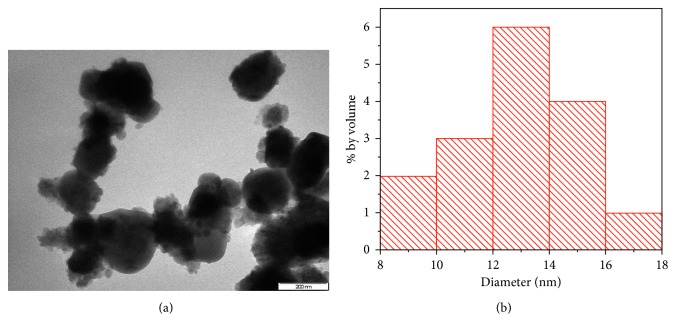
TEM image of (a) EB@TiO_2_ composite; (b) particle size distribution.

Modification of eggshell with titanium dioxide was achieved in two steps. In the first step, eggshells were prepared according to the procedure described by Onwubu et al. [21]. Eggshells were disinfected by storing them in a diluted solution of household sodium hypochlorite for six hours. Subsequently, eggshells were vacuum-dried for ± 6–9 min at 250°C. Thereafter, 30 g of the dried eggshell was placed in a 500 ml stainless jar (inner diameter of 100 mm), together with 50 stainless steel balls of 10 mm diameter, and dry-milled in a planetary ball mill (Retsch® PM 100) at 400 rpm for 20 minutes. The collected powder was sieved to a particle size of ≤ 25 *µ*m using a mechanical sieving shaker (Retsch AS 200, Germany). In the next step, 20 g of the fine eggshell powder obtained in step 1 was modified by adding 5 g of anatase titanium dioxide (≤15 *µ*m). The mixture was subsequently ball-milled for 200 min to obtain eggshell-titanium dioxide composite (EB@TiO_2_). The particle size distribution of the composite measured using high-resolution transmission microscope (TEM-Philips CM 120 model) at 120 kV and image J software (National Institute of Health USA, http://imagej.nih.gov./ij) was found to be 14 nm (Figure 2).

### 2.2. pH Measurement

The pH and buffering characteristics of the EB@TiO_2_ (tested samples) and the respective toothpastes, brand names, and manufacturers are given in Table 1. The pH of each of the samples in deionized water as well as in 0.01 mol·L^−1^ hydrochloric acid (HCI) were measured by placing 1.5 g of each samples in a beaker containing 50 mL deionized and HCI, respectively. The solution was constantly agitated at low speed of 600 rpm for 30 min. A pH meter (Starter 300, Ohaus Incorporation USA) equipped with temperature sensor was used to record changes in the pH reading. Before testing the samples, the pH was calibrated using tested buffer of known pH solutions.

### 2.3. Preparation of Tooth Enamel Specimens

Twenty freshly collected bovine enamel anterior teeth were used to evaluate the acid resistant properties of EB@TiO_2_. The collected teeth were subsequently cleaned and disinfected in 10% chloroxylenol solution. Enamel specimens measuring 5 mm × 5 mm × 3 mm was prepared after cutting off the roots using a low-speed diamond saw under water cooling conditions. Before subjecting the specimens to acidic condition, the specimens were embedded in a resin (FT 160; AMT composite), and a silicone mold (Blue mold; Agar scientific) was used to make a mounting base. The embedded specimens were randomly assigned into five groups (*n*=4) and were then exposed to 0.01 mol·L^−1^ HCI (pH = 2) for 30 min as follows: Group 1: unexposed tooth enamel; Group 2: exposed tooth enamel + HCI; Group 3: exposed tooth enamel + HCI + Colgate; Group 4: exposed tooth enamel + HCI + Sensodyne; and Group 5: exposed tooth enamel + HCI + EB@TiO_2_.

After 30 min of acid exposure, the specimens were rinsed in deionized water for 30 s and blot-dried.

### 2.4. Atomic Force Microscopy (AFM) Analysis

AFM (Nanoscope; Bruker) was used to analyze the mean square roughness (R_rms_) values of the specimens. The instrument was set in contact mode with a scanning size of 10 *µ*m and a scan rate of 2.394 Hz. For each specimen, 5 different measurements of the *R*_rms_ values were made. The average measured *R*_rms_ values were then used for the statistical analysis.

### 2.5. Field-Scanning Electron Microscope Evaluation of the Specimens

The surface of the exposed and unexposed specimens was characterized using scanning electron microscope (FESEM; Carl Zeiss). As a proxy measure, a specimen from each group was analyzed microscopically.

### 2.6. Raman Spectroscopy Analysis of Specimens

The changes in the mineral content of the specimens were observed using a Raman (Perkin Elmer *precisely* Raman-station 400). The Raman analysis was done on the all the samples with the laser power set at 70 mW, exposure every 10 seconds for 3 seconds at a time. Five different measurements were done for each samples and the average was used for statistical analysis.

### 2.7. Statistical Analysis

The mean roughness (*R*_rms_) value of the specimens was evaluated with 1-way analysis of variance (ANOVA) by means of statistical software (IBM SPSS Statistics v25; IBM Corp.), followed by Bonferonni correction and the significance level was set at *α*=0.05. For Raman analysis, the base line correction was estimated by the polynomial fitting method, and the peak area and height were determined from the Gaussian plot using Origin Pro software (OriginLab Corporation v 8).

## 3. Results

### 3.1. Atomic Force Microscopy (AFM)

The 1-way ANOVA, mean, standard deviation, and standard error results are shown in Table 2. A notable statistical difference was observed in the mean surface roughness (*R*_rms_) for the unexposed and exposed specimens (*P* < 0.05).

**Figure 3:**
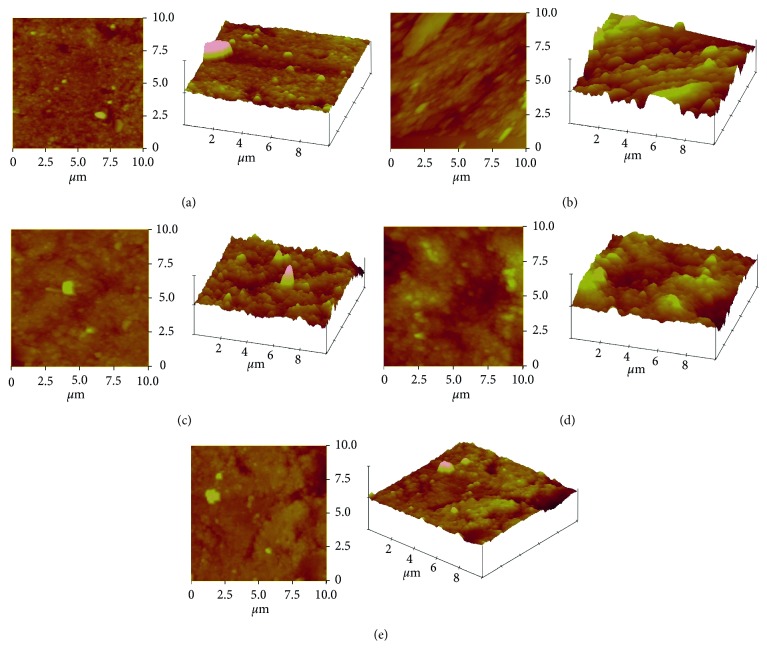
AFM profile of (a) unexposed tooth; (b) after exposure to HCI; (c) after exposure to HCI + Colgate toothpaste; (d) after exposure to HCI + Sensodyne; (e) after exposure to HCI + EB@TiO_2_.

Comparing the unexposed specimens (group 1) with specimens exposed to HCI alone (group 2), a significant difference were measured (*P* < 0.05). The unexposed specimens had the lowest mean *R*_rms_ (32.6 ± 16.3 nm) values while specimens exposed to HCI alone had the highest value (101.9 ± 18.0 nm). No significant differences were measured for the unexposed specimens and the specimens exposed in groups 3, 4, and 5, respectively (*P* > 0.05). AFM micrograph shown in Figure 3 further illustrates the surface profile of the specimens. The surface roughness appeared more pronounced for Figures 3(b)–3(d) when compared against Figures 3(a) and 3(e).

### 3.2. Field-Scanning Electron Microscope Observation of Specimens

**Figure 4:**
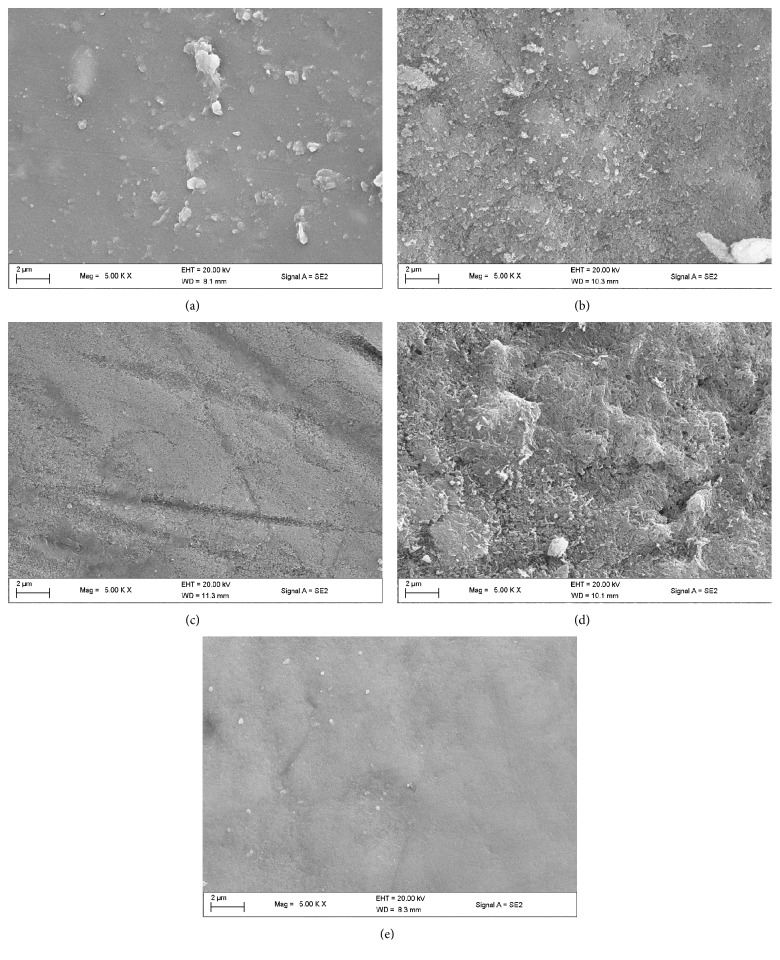
FESEM images of (a) unexposed tooth; (b) after exposure to HCI; (c) after exposure to HCI + Colgate toothpaste; (d) after exposure to HCI + Sensodyne; (e) after exposure to HCI + EB@TiO_2_.

The FESEM images of the specimens are shown in Figure 4. The images revealed surface differences between the unexposed specimens, exposed tooth alone, and the test groups (EB@TiO_2_ and commercial toothpastes). While using the unexposed specimen to benchmark the demineralization of the exposed sample groups, the images in Figures 4(b) and 3(d) visibly showed evidence of the prismatic destruction of the hydroxyapatites which suggest erosion of tooth specimens exposed to acid alone and the Sensodyne toothpaste. In contrast, the tooth specimen exposed to acid in the presence of both EB@TiO_2_ and Colgate showed less evidence of the prismatic destruction of the enamel (Figures 4(c) and 3(e)).

### 3.3. Raman Spectroscopy

**Figure 5:**
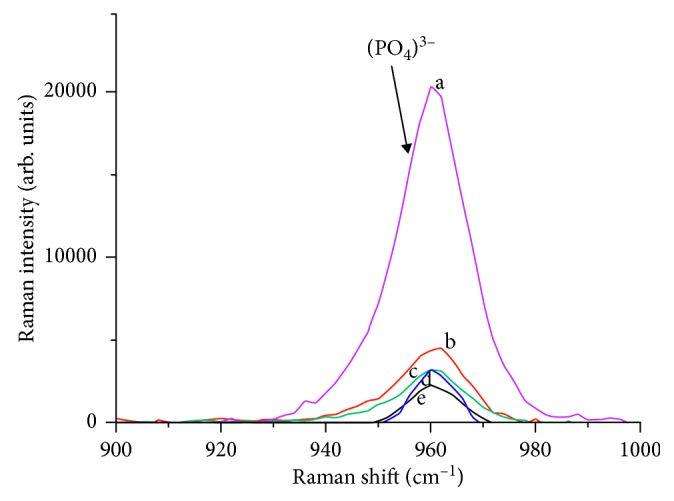
Raman spectrum obtained for tooth samples (a) expose to HCI alone; (b) expose to HCI + Sensodyne; (c) exposed HCI + Colgate toothpaste; (d) expose to HCI + EB@TiO_2_; (e) unexposed tooth.

Furthermore, the changes in the position of phosphates (V_3_ (PO_4_^3−^)) peaks for the unexposed and exposed specimens are given in Figure 4. In the unexposed specimens, the peak was only slightly prominent (Figure 5). On the other hand, the peaks for the specimen exposed in HCI alone (Figure 5) were distinctly prominent to a considerable height (Table 3). Moreover, only slight changes were noticed in the peaks of the specimens exposed to sample groups 3 (Figure 5), 4 (Figure 5), and 5 (Figure 5), respectively.

## 4. Discussion

The purpose of this study was to examine the protective effect of a modified eggshell-titanium dioxide composite (EB@TiO_2_) in comparison with some commercial toothpastes against erosive acids. In line with suggested techniques in the literature [22–24], AFM was used to characterize and measure the changes in the surface roughness pre- and postexposure to HCI. The average mean square roughness values (*R*_rms_) measured were used for statistical analysis. The *R*_rms_ is the standard deviation height obtained from the AFM images (Figure 3) in areas of 30 × 30 *µ*m^2^ with a resolution of 256 × 256 pixels. The *R*_rms_ data demonstrated that exposure of tooth enamel to HCI significantly affected the surface roughness (*P* < 0.05).

More so, the *R*_rms_ values measured for the enamel tooth specimens exposed to HCI alone group were significantly higher than the unexposed tooth enamel (*P* < 0.05). This strongly confirmed that the exposure of enamel to acidic substances leads to enamel demineralization. Whilst tooth enamel comprises mostly of calcium (Ca^2+^), phosphates (PO_4_^3+^), and hydroxide (OH^−^), the enamel is constantly in equilibrium with the surrounding saliva and enamel crystals. In support of Shellis et al. [6], the exposure of the tooth enamel to HCI alone may cause enamel to release more ions to the environment to attain a new state of equilibrium. Consequently, and consistent with Lussi and Carvalho [7], the acidic condition exacerbated the process leading to enamel demineralization (Figure 4(b)).

This notwithstanding, the *R*_rms_ values measured for the enamel tooth specimen exposed to Colgate, Sensodyne, and EB@TiO_2_ were not different from the unexposed tooth (*P* > 0.05). In light of these, it can be inferred that the toothpastes (Colgate and Sensodyne) and test group (EB@TiO_2_) were protective against enamel demineralization. This supports the notion that the sampled toothpastes are effective and accessible vehicles to improve enamel resistant against erosive oral environment [9]. In comparing the protective effect of the sampled toothpastes samples against EB@TiO_2_, the *R*_rms_ values with the highest mean was for Sensodyne and the lowest for EB@TiO_2_ (Table 2). This could have been attributed to the presence of Ca and or titanium ions in the exposed tooth in the presence of EB@TiO_2_. More so, and given the differences in the mean *R*_rms_ values between EB@TiO_2_ and Colgate toothpaste, the observed differences appear to reflect the buffering capacities of the 2 groups (Table 1). This strongly supports Caneppele et al. [25] that the buffering capacity of acidic concentration is critical in erosive potential of acidic substance, which may influence the dissolution rate of the enamel. Equally, the differences in the *R*_rms_ values between EB@TiO_2_ and Colgate toothpastes may also be associated with the modification of the calcium carbonate constituents of eggshell with titanium dioxide. Hence, the tested hypothesis is partially accepted as the EB@TiO_2_ composite effectively protected the tooth enamel against erosion.

Furthermore, the Raman spectrum of the exposed and unexposed tooth enamel observed at a peak of 960 cm^−1^ could be attributed to the symmetrical stretching of the tetrahedron oxygen atoms that surrounds the phosphorus atoms [26, 27]. This result is consistent with Ionita [28] that Raman spectrum of healthy enamel and demineralized tooth is observed at a well-defined peak at 959 cm^−1^ vibrations. Moreover, the highest-Raman spectrum demineralization intensity observed for the exposed tooth alone in HCI was nearly 5-times greater than the intensity measured for the unexposed tooth (Figure 5). In addition, He et al. [29] and Targino et al. [30] theorize that the mineral intensity measured in Raman could be a factor that determines the rate of demineralization of tooth enamel. Significantly, the Raman intensity measured for EB@TiO_2_ was comparable to those measured for Colgate toothpaste; however, it was lower than the sampled Sensodyne toothpaste (Figure 5). This observation is consistent with the results of Joiner et al. [16] which show that the addition of calcium and or calcium containing materials into toothpaste improves its resistant against acidic erosion on tooth enamel.

In light of the above findings, the researchers plan to further examine the remineralization potential of EB@TiO_2_, particularly in the repair of damaged tooth (enamel and dentine). These studies would help establish the suitability of EB@TiO_2_ in the maintenance of oral health particularly as a desensitising toothpaste.

## 5. Conclusion

In conclusion, this study has demonstrated that toothpastes provide protection to the tooth enamel against erosive substance. Notably, the study has shown that EB@TiO_2_ offer better protective covering to the enamel against acid attack.

## Figures and Tables

**Table 1:** Toothpastes used and their respective pH and buffering characteristics.

Toothpastes	Brand name	Manufacturer	pH in deionized water	Buffering capacity *Β* (mmol 2^−1^ pH^−1^)
Colgate	CALCI^TM^—SEAL protection	Colgate-Palmolive Co	9.61	6.6 ± 02.9
Sensodyne	Rapid relief	GlaxoSmithKline	7.41	2.3 ± 0.02
EB@TiO2	N/A	Researcher	9.31	7.3 ± 0.11

**Table 2:** Mean surface roughness, standard deviation, standard error, and ANOVA.

Groups	*N*	Mean ± SD	Std. error	95% confidence interval for mean		*P*	Post hoc bonferroni test
				Lower bound	Upper bound		*P*
Unexposed tooth	4	32.6 ± 16.3 nm	8.1	6.7077	58.5478	0.021	0.02^1,2^
Exposed tooth + HCI	4	101.9 ± 18.0 nm	9.0	73.1958	130.5232		0.295^1,5^
Exposed tooth + HCI + Colgate	4	65.2 ± 29.0 nm	14.5	19.0787	111.4123		0.992^1,3^
Exposed tooth + HCI + Senosdyne	4	83.1 ± 33.7 nm	16.8	29.4663	136.6767		0.159^1,4^
Exposed tooth + EB@TiO_2_	4	57.2 ± 29.6 nm	14.8	10.0747	104.3583		1.00^1,5^

Superscript numbers indicate significant differences between the sample groups (ANOVA, *P* < 0.05).

**Table 3:** Peak analysis as determine by the Gaussian plot.

Sample groups	Peak parameters	
	Height	Area
Unexposed tooth	2454.2	32769.3
Tooth exposed to HCI	18853.3	345765.1
Tooth exposed to HCI + Colgate toothpaste	3956.9	52589.4
Tooth exposed to HCI + Sensodyne	4270.3	73357.7
Tooth exposed to HCI + EB@TiO_2_	2965.9	49185.4

## Data Availability

The data used to support the findings of this study are included within the article.
