# Corrigendum to “An In Situ Evaluation of the Protective Effect of Nano Eggshell/Titanium Dioxide against Erosive Acids”

**DOI:** 10.1155/2019/7209168

**Published:** 2019-03-10

**Authors:** Stanley Chibuzor Onwubu, Phumlane Selby Mdluli, Shenuka Singh, Sanele Nyembe, Rookmoney Thakur

**Affiliations:** ^1^Dental Sciences, Durban University of Technology (DUT), Durban, South Africa; ^2^Chemistry, Durban University of Technology (DUT), Durban, South Africa; ^3^Discipline of Dentistry, University of KwaZulu-Natal (UKZN), Durban, South Africa; ^4^DST/Mintek Nanotechnology Innovation Centre, Advanced Materials Division, Mintek, Private Bag X3015, Randburg, Johannesburg 2125, South Africa; ^5^Public Management and Economics, Durban University of Technology (DUT), Durban, South Africa

In the article titled “An In Situ Evaluation of the Protective Effect of Nano Eggshell/Titanium Dioxide against Erosive Acids” [1], the affiliation of Dr. Rookmoney Thakur was incorrect. The corrected authors' affiliations are shown above.
